# Subjective and objective working memory deficits in the post-acute phase of COVID-19 in a clinical trial population

**DOI:** 10.1016/j.bbih.2026.101278

**Published:** 2026-06-04

**Authors:** Sofie Buer, Bjørn I. Hagen, Arne Søraas, Richard A. White, Anners Lerdal, Anders B. Nygaard, Jan Stubberud

**Affiliations:** a Department of Research, Lovisenberg Diaconal Hospital, Oslo, Norway; b Department of Psychology, University of Oslo, Oslo, Norway; c Department of Microbiology, Oslo University Hospital, Oslo, Norway; d Faculty of Medicine, University of Oslo, Oslo, Norway; e Uppsala University, Uppsala, Sweden

**Keywords:** Neuropsychology, Executive functioning, Working memory deficits, Post-COVID-19 condition, Cognitive functioning, Neuropsychological assessment

## Abstract

**Importance:**

Cognitive difficulties are commonly reported following COVID-19, yet the relationship between subjective and objective working memory (WM) deficits remains unclear. Examining both types of measures may improve understanding of cognitive complaints in individuals with persistent post-COVID-19 symptoms.

**Objective:**

This cross-sectional study aimed to describe WM deficits in a clinical trial population with self-reported persistent cognitive difficulties >6 months following COVID-19, compare the frequency of scores exceeding clinical cutoffs on subjective versus objective measures, and explore how these frequencies vary according to demographic, clinical, and selected COVID-19-related characteristics.

**Methods:**

We used baseline data from a preregistered randomized controlled trial (Hagen et al., 2022). Participants (N = 129) with perceived executive function deficits aged 26-65 years, and at least one prior laboratory-confirmed SARS-CoV-2 infection were included. Subjective WM was assessed using the Behavior Rating Inventory of Executive Function-Adult Version, Working memory subscale. Objective WM was measured using Digit Span from the Wechsler Adult Intelligence Scale and Spatial Working Memory from Cambridge Neuropsychological Test Automated Battery. Participants also completed questionnaires on anxiety and depression, fatigue, and insomnia.

**Results:**

Participants more frequently exceeded the clinical threshold (T ≥ 65) for subjective WM deficits than for objective WM deficits (1.5 *SD* below normative mean). Higher levels of anxiety and depression were associated with a higher frequency of exceeding clinical thresholds for subjective, but not objective, WM deficits.

**Conclusion:**

In this selected clinical trial population of individuals with persistent self-reported cognitive difficulties following COVID-19, subjective WM deficits were more frequent than objective WM deficits. The findings reveal a low association between subjective and objective WM deficits in the post-acute phase of COVID-19 recovery and indicate that non-cognitive factors, particularly anxiety and depression symptoms, may be relevant when evaluating subjective WM complaints following COVID-19.

## 1 Introduction

At least 10% of global COVID-19 cases are projected to endure persistent and long-lasting symptoms (Altmann et al., 2023; Ellingjord-Dale et al., 2024), with many symptoms remaining unresolved even years following the initial SARS-CoV-2 infection (Davis et al., 2023; Fernandez-de-las-Peñas et al., 2024; O'Mahoney et al., 2023). Importantly, cognitive deficits, primarily characterized by lingering difficulties in attention and executive function (EF), including working memory (WM), are common symptoms and persist into the post-acute phase (Becker et al., 2023; Buer et al., 2024; Douaud et al., 2022; Ellingjord-Dale et al., 2024; Hampshire et al., 2024). This underscores a significant health concern emerging in the aftermath of the pandemic.

Working memory, a central aspect of EF, refers to the ability to hold and manipulate task-relevant information during ongoing cognitive activity (Baddeley, 1992; Miyake et al., 2000; Snyder et al., 2021). WM deficits following COVID-19 may compromise daily functioning and quality of life (Cui et al., 2024).

WM can be assessed using subjective self-report questionnaires and objective neurocognitive performance tests. Previous studies have reported both self-reported WM difficulties and objective cognitive deficits following COVID-19, including deficits in attention and WM (Arbula et al., 2024; Bland et al., 2024; Buer et al., 2024; Hampshire et al., 2021). Objective deficits have also been linked to structural brain alterations in regions relevant for memory function (Douaud et al., 2022). Thus, both subjective and objective evaluations may capture alterations in WM.

However, objective WM deficits do not necessarily correlate with subjective WM complaints in the post-acute phase of COVID-19 (Arbula et al., 2024; Becker et al., 2023). Similar discrepancies between rating-based and performance-based EF measures have been observed across clinical populations (Haugen et al., 2021; Ingulfsvann Hagen et al., 2023; Øie et al., 2022), with subjective measures often reflecting everyday functioning in the presence of everyday distractions and being more sensitive to contextual or psychological factors, while objective measures capture performance under structured testing conditions (Orfei et al., 2022; Van Aken et al., 2023). Factors such as emotional distress, fatigue, sleep problems, metacognitive capacity, and contextual demands may therefore contribute to discrepancies between subjective and objective WM measures (Gerst et al., 2017; Nin et al., 2022; Snyder et al., 2021; Soto et al., 2020; Toplak et al., 2013).

To date, very few studies have combined subjective and objective measures to evaluate WM in this patient population. Further, it remains unclear whether clinically relevant factors commonly reported in post-COVID-19 conditions, such as anxiety and depression, fatigue, and sleep problems (Kirchberger et al., 2023; Poletti et al., 2022; Reid et al., 2024), are related to discrepancies between subjectively and objectively measured WM deficits. By focusing specifically on WM and applying clinical cutoffs to both subjective and objective measures within the same clinical trial population, the present study extends prior work by examining clinically relevant discrepancies in WM functioning after COVID-19.

The aim of the current cross-sectional study was to (1) examine the severity of WM deficits in a clinical trial population with self-reported persistent cognitive difficulties following COVID-19, (2) identify individuals who exceed clinical thresholds on subjective and objective WM measures, and (3) compare the frequency of clinically significant subjective and objective WM deficits and examine whether these classifications vary according to demographic, clinical, and selected COVID-19-related characteristics, including anxiety and depression, fatigue, and insomnia.

## 2 Methods

The current study uses data from a baseline assessment for a preregistered randomized controlled trial (NCT05494424) investigating the effectiveness of a cognitive rehabilitation intervention in post-COVID-19 condition (Hagen et al., 2022). The research was completed in accordance with the Helsinki Declaration and has been approved by the Regional Research Ethics Committee (2022/024), South-Eastern Norway. All participants have provided electronically signed informed consent upon participation. The data were collected at Lovisenberg Diaconal Hospital (Norway) between October 2023 and April 2024.

Prior to invitation, one or more confirmed positive real-time polymerase chain reaction (rt-PCR) test(s) in any accredited Norwegian clinical microbiology laboratory was required. The data was obtained from the Norwegian Health Register 'MSIS' (Reporting System for Infectious Diseases). Data on SARS-CoV-2 vaccination status were obtained from the Norwegian national mandatory registry on vaccination (SYSVAK) (Søraas et al., 2022).

Participants were invited through their ongoing participation in the Norwegian COVID-19 Cohort Study. Participants were recruited from across Norway using social media, personal invitations, and media coverage starting March 27, 2020 (Søraas et al., 2021). A random sub-sample (*n* = 3247) was generated based on the following criteria: (1) confirmed SARS-CoV-2 infection, (2) over 18 years old, under 65 years old, (3) still actively participating in the Norwegian Cohort Study indicated by completion of the latest follow-up form. In the invitation participants were asked to respond if they were experiencing persistent cognitive deficits (>2 months) following SARS-CoV-2 infection. Out of the individuals invited, 493 (15%) responded and 345 (11%) signed an online consent form.

To be included, participants needed to self-report with a “yes” to cognitive difficulties during a customized telephone interview based on the following two screening questions: (1) Have you experienced difficulties with concentration, memory, or decision-making that have lasted for more than 2 months following COVID-19? (2) Do any of these cognitive difficulties affect your daily activities? These cognitive complaints had to be subjectively attributed to COVID-19, and inclusion required a 'yes' response to both screening questions. Out of the 345 individuals who signed the online consent form, 104 potential participants were excluded during the customized telephone interview because they did not self-report subjective cognitive difficulties following COVID-19 and therefore did not meet the inclusion criteria.

**Fig. 1:**
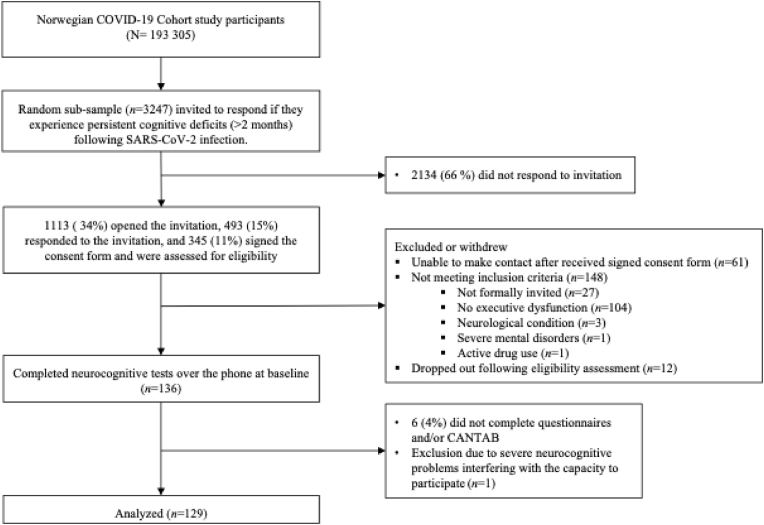
Patient flow overview.

All eligibility evaluations (*n* = 284) were conducted or trained to conduct eligibility evaluations by a clinical psychologist. In addition to self-reported post-COVID-19 cognitive difficulties, exclusion criteria included ongoing alcohol- or substance abuse, premorbid insult and/or comorbid neurological disease, severe neurocognitive problems interfering with the capacity to participate (scoring <10 on the MiniMoCa) (Wong et al., 2015), sensory disorders affecting cognitive assessment, schizophrenia or bipolar disorder with mood congruent psychotic features, lack of proficiency in Norwegian, and being previously enrolled in a cognitive rehabilitation trial (Hagen et al., 2022). After completing the screening interview, 136 participants were included in the study, and 129 completed all baseline measures (see Fig. 1).

## 3 Measures

All participants were tested and interviewed on a large battery of measures (Hagen et al., 2022), and only selected data are included in the current study. After eligibility evaluations, participants self-reported their highest level of education and comorbidities during a customized 30- to 40-min telephone interview. At the end of the interview, another appointment was scheduled to complete a 30-min cognitive assessment over the phone. These cognitive tests were administered in a fixed order by the same clinical psychologist. All other cognitive measures, including all self-report questionnaires were completed remotely using unique links emailed to the participants.

The Hospital Anxiety and Depression Scale (HADS) is a widely used self-report questionnaire designed to measure symptoms of anxiety and depression in a general medical population (Zigmond and Snaith, 1983). It consists of 14 items, with seven items addressing symptoms of anxiety and seven addressing depression. Each item is rated 0 to 3 on a Likert scale, where higher scores indicate more severe symptoms. The HADS has demonstrated adequate test-retest reliability and factor structure and performs satisfactorily in assessing the severity and caseness of anxiety disorders and depression in the general population (Bjelland et al., 2002). Scores below 7 indicate non-cases, 8-10 indicate mild symptoms, 11-14 indicate moderate symptoms, and 15-21 indicate severe symptoms (Stern, 2014).

The Insomnia Severity Index (ISI) assesses the nature, severity and impact of insomnia, and is a reliable and valid measure for detecting insomnia in the general population, as well as being sensitive to treatment response in clinical patients (Morin et al., 2011). The ISI is a 5-point Likert scale in which participants rate each item from 0 indicating no problem to 4 indicating a very severe problem, yielding a total score ranging from 0 to 28. Cutoff scores are as follows: 0-7 indicate no insomnia, 8-14 indicate subthreshold insomnia, 15-21 indicate moderate insomnia, and 22-28 indicate severe insomnia (Morin et al., 2011).

The 7-item version of the Fatigue Severity Scale (FSS) was used to assess fatigue. Each item is scored on a 7–point Likert scale ranging from 1 (“strongly disagree”) to 7 (“strongly agree”). The FSS evaluates characteristics of fatigue, focusing on how it interferes with various aspects of daily functioning (Johansson et al., 2014; Lerdal et al., 2011). It is widely used to measure fatigue in individuals with chronic illnesses. The mean sum cutoff scores applied are <4 indicating no to very low fatigue, ≥4 and < 5 indicating moderate fatigue and ≥5 indicating severe fatigue (Lerdal et al., 2011).

### 3.1 Subjective working memory

Self-reported WM was measured using the Behavior Rating Inventory of Executive Function - Adult Version (BRIEF-A), a 75-item standardized questionnaire, comprising nine subscales. These contribute to two index scores, the Behavioral Regulation Index (BRI) and the Metacognition Index (MI), as well as a Global Executive Composite (GEC). While BRIEF-A captures a broad range of EFs, only the Working Memory subscale from the MI is emphasized in the present study. Participants rated the frequency of specific problematic behaviors over the past six months using a three-point Likert scale (never = 1; sometimes = 2; or often = 3). Raw scores were transformed into age-corrected *T*-scores (*M* = 50; *SD* = 10) based on U.S. normative data. The clinical cutoff score is *T* ≥ 65, with recommended validity scale cutoffs of Negativity >6, Infrequency >3, and Inconsistency >8 (Roth et al., 2005). Participants exceeding more than one validity scale cutoff were excluded (Roth et al., 2005). BRIEF-A has excellent psychometric properties with one-month test-retest reliability ranging from.82 to 0.93 for all subscales (Roth et al., 2005; Waid-Ebbs et al., 2012).

### 3.2 Objective working memory

The following neurocognitive tests were included as objective measures of WM; (1) Digit Span Forward from the Wechsler Adult Intelligence Scale - Fourth Edition (WAIS-IV; Wechsler, 2008) was used to differentiate between potential deficits in attention and WM, (2) Digit Span Backward from the WAIS-IV, and (3) Spatial Working Memory (SWM) test from the Cambridge Neuropsychological Test Automated Battery (CANTAB) (Pickett, 2021; Stavem et al., 2022). The clinical threshold for Digit Span Forward and Backward is defined as an age-corrected scaled score of 5.5 or below (1.5 *SD* below average), and for SWM, as a z-score 1.5 *SD* below the age-expected average (Lezak et al., 2012). Digit span forward was considered a measure of attention, while digit span backwards a measure of WM. The SWM test evaluates spatial WM and strategy, commonly used across neurological and psychiatric conditions (Karlsen et al., 2022; Stavem et al., 2022; Wechsler, 2008). All tests were completed in Norwegian. The Digit Span from the WAIS-IV was performed over the telephone, but all tests from the CANTAB battery was completed digitally using a unique remote link sent by email.

### 3.3 Estimate of verbal intelligence

The Similarities subtest from the WAIS-IV measures verbal concept formation as part of the verbal comprehension index and were used as an estimate of verbal intelligence (Wechsler, 2008).

### 3.4 Statistical analysis

All cases that completed the baseline assessment (*n* = 129) were included in the analysis. Data were described using mean and standard deviations (*SD*) for continuous variables and with counts and percentages for categorical variables. For the main analysis, multiple contingency tables were produced to examine whether the frequency of scoring above or below predefined clinical thresholds on subjective and objective measures of attention and working memory varied according to selected covariates. That is, for each of the four binary outcomes, contingency tables were produced against the categorical variables: time since first SARS-CoV-2 infection, self-reported SARS-CoV-2 re-infections, maximum number of days bedridden, severity of post-COVID-19 fatigue, severity of insomnia and symptom levels of anxiety and depression, as well as number of comorbidities. For each of the 32 contingency tables, Fisher's exact test was applied. A false discovery rate correction was applied to adjust for the 32 tests performed. For investigating potential sex differences across the subjective and objective measures of WM deficits, Fisher's exact test was also applied here. All data, analysis code, and research materials are available upon request. All statistical analyses were performed using R Statistical Software (version 4.2.2 R Foundation for Statistical Computing, Vienna, Austria) using a 0.05 significance level.

## 4 Results

A total of 136 participants with at least one confirmed positive SARS-CoV-2 infection were included at baseline following neurocognitive testing via telephone. Six participants (4%) were excluded due to incomplete baseline data. The demographic characteristics of the final sample of 129 participants are outlined in Table 1. The mean age was 48 (*SD* = 9) years; 77% were women. Based on the date of their first positive SARS-CoV-2 infection, 12 % were presumed to have had the initial virus strain (variants of B.1), 10% had the Alpha variant, and 77% had the Delta variant (Fig. 1, Supplementary) (Lyngstad, 2022).

**Table 1:** Demographics and clinical characteristics of the study sample at baseline (*N* = 129).

Characteristics	Range	Mean (SD)/Count n (%)
Sex		
Female		99 (77)
Male		30 (23)
Age	26-65	48 (9)
Ethnicity		
European		123 (98)
Asian		1 (0.8)
Other		2 (1.6)
Unknown		3
Education (years)	10-23	16.88 (2.45)
Verbal IQ^a^	5-18	10.69 (2.61)
Comorbid chronic disorders^b^		
None		49 (38)
One		44 (34)
Two		19 (15)
Three or more chronic comorbidities		16 (13)
Symptoms of anxiety and depression	0-30	12 (7)
Fatigue levels	1-7	5.06 (1.55)
Insomnia	0- 25	10.6 (6)
Time since first SARS-CoV-2 infection		
Less than 2 yrs.		34 (26)
2 yrs – 2 ½ yrs.		62 (48)
2 ½ yrs. – 3 yrs.		14 (11)
Over 3 yrs.		19 (15)
Hospitalized due to COVID-19 (self-report)		
Yes		8 (6.3)
No		120 (93.8)
Missing		1
Maximum number of days bedridden in the acute phase of SARS-COV-2^c^		
None		18 (16)
1-6 days		67 (60)
7-13 days		22 (20)
Two weeks or more		5 (4.5)
Unknown		17
Vaccinated prior to first SARS-CoV-2 Infection		93 (72)
Number of Self-reported SARS-CoV-2 infections		
One		32 (25)
Two		46 (36)
Three or more		50 (39)
Unknown		

a The Similarities subtest; WAIS-IV.

b Previous concussion with a history of persistent post-concussive symptoms, but no current persistent post-concussive symptoms, heart disease, ADHD/ADD, diabetes, history of anxiety, history of depression, learning difficulties, hypothyroidism, morbid obesity, and other illnesses such as high blood pressure, lyme disease, high-functioning autism, bipolar disorder without mood congruent psychotic features, a history of PTSD, and history of cancer treatment. One participant with a benign brain tumor was included in the "Other" category after reporting no cognitive impairments following diagnosis.

c A proxy for symptom severity in the acute phase of SARS-CoV-2.

### 4.1 Frequency above clinical thresholds in the study population

Most participants scored above the clinical cutoff (*T* ≥ 65) on the BRIEF-A Working Memory subscale (69 %) (Table 2 and eSupplementary Table 1). Approximately 4% scored 1.5 *SD* below the age-expected mean for the two objective measures on WM (Table 2). Only 3.1% scored 1.5 *SD* below the normative mean for the objective measure for attention (Table 2). No statistically significant differences emerged between the sexes (eSupplementary Figure 2 and eSupplementary Figure 3).

**Table 2:** Descriptives of objective and subjective working memory.

Working memory	*n*	Mean (*SD*)	Range	% over cutoff^a^
Performance-based attention				
Digit span forwards (WAIS-IV)	128	11.98 (3.16)	4-18	3.1
Performance-based WM				

Digit span backwards (WAIS-IV)	128	10.30 (2.65)	5-17	3.9
Spatial WM (CANTAB; SWM)	122	0.16 (1.36)	−1.88 – 2.33	4.1
Self-reported WM				

Working Memory (BRIEF-A)	128	69.59 (11.14)	43-94	68.75

a % over cutoffs are estimated based on 1.5 *SD* below normative mean for WAIS-IV subtests and CANTAB: SWM, and 1.5 *SD* above U.S. normative mean for BRIEF-A.

### 4.2 Frequency above clinical threshold according to covariates

Objective and subjective WM deficits were not significantly more prevalent in any of the defined periods of time since first SARS-CoV-2 infection. Most participants (*n* = 93) were vaccinated prior to their first infection, and unvaccinated participants (*n* = 35) did not show significantly more frequent objective or subjective WM deficits. Similarly, self-reporting more than one SARS-CoV-2 infection and symptom severity in the acute phase of SARS-CoV-2 as indicated by maximum number of days bedridden did not display significantly more frequent objective or subjective WM deficits (Table 3).

**Table 3:** Comparison of subjective and objective working memory deficits with COVID-19 specific covariates.

Clinical Variables	*n*	Performance-based attention	*n*	Performance-based WM	*n*	Performance-based Spatial WM	*n*	Self-reported WM
IN TOTAL SAMPLE	129	3.1%	128	3.9%	122	4.1%	128	68.8%
Time since first SARS-CoV-2 infection								
Less than 2 yrs	34	2.9%	34	2.9%	33	3.0%	34	64.7%
2 yrs to 2 ½ yrs	62	1.6%	62	4.8%	59	0.0%	62	72.6%
2 ½ yrs to 3 yrs	14	0.0%	14	7.1%	13	23.1%	13	84.6%
Over 3 yrs	18	11.1%	18	0.0%	17	5.9%	19	52.6%
P-value^a^		0.188		0.688		0.004		0.231
FDR Q-value		0.656		1.000		0.064		0.672
Hospitalized in the acute phase of COVID-19^b^								
No	119	3.4%	119	4.2%	114	4.4%	120	69.2%
Yes	8	0.0%	8	0.0%	7	0.0%	7	71.4%
P-value^a^		1.000		1.000		1.000		1.000
FDR Q-value		1.000		1.000		1.000		1.000
Vaccinated prior to first SARS-CoV-2 infection								
Not vaccinated	35	5.7%	35	0.0%	32	9.4%	35	60.0%
Yes	93	2.2%	93	5.4%	90	2.2%	93	72.0%
P-value^a^		0.301		0.322		0.112		0.205
FDR Q-value		0.757		0.757		0.656		0.656
Self-reported SARS-CoV-2 infections								
One	32	6.2%	32	3.1%	31	3.2%	32	68.8%
Two	45	2.2%	45	4.4%	42	4.8%	45	62.2%
Three or more	50	2.0%	50	4.0%	48	4.2%	50	76.0%
P-value^a^		0.559		1.000		1.000		0.338
FDR Q-value		0.945		1.000		1.000		0.757
Maximum number of days bedridden in the acute phase of SARS-COV-2								
None	17	0.0%	17	5.9%	18	11.1%	18	44.4%
1-6 days	67	1.5%	67	6.0%	64	1.6%	67	70.1%
7-13 days	22	4.5%	22	0.0%	19	5.3%	21	76.2%
Two weeks or more	5	0%	5	0.0%	5	0.0%	5	80.0%
P-value^a^		0.638		0.729		0.171		0.144
FDR Q-value		0.972		1.000		0.656		0.656

a Fisher's exact test.

b Hospitalization status was based on self-report with a small number of hospitalized participants.

The majority of the clinical trial sample had not been hospitalized in the acute phase of COVID-19 (*n* = 120). Hospitalized participants (*n* = 8) did not show significantly more objective or subjective WM deficits (Table 3). Moreover, hospitalized and non-hospitalized participants displayed similar patterns on self-reported executive functioning across all scales and were not limited to WM deficits (eSupplementary Figure 4).

The severity of insomnia, fatigue, and symptoms of anxiety and depression did not significantly impact the frequency of objective WM deficits (Table 4). Subjective WM deficits were significantly more frequent among participants with more severe fatigue; 77.2% of participants with severe fatigue exceeded the clinical cutoff on the BRIEF-A Working Memory subscale (*n* = 79, *p* < .05). However, after the false discovery rate correction was applied to adjust for the 32 tests performed, fatigue was no longer significant (p < .160). Subjective WM deficits were significantly more frequent among participants with higher levels of anxiety and depression symptoms; 90% of participants with severe anxiety and depression symptoms exceeded the clinical cutoff on the BRIEF-A Working Memory subscale (*n* = 30, *p* < .001; Table 4). The association between self-reported WM deficits and anxiety and depression symptoms remained significant after false discovery rate correction.

**Table 4:** Comparison of subjective and objective working memory deficits with covariates of fatigue levels, insomnia and mental health.

Clinical Variables	*n*	Performance-based attention	*n*	Performance-based WM	*n*	Performance-based Spatial WM	*n*	Self-reported WM
Fatigue								
No to very low fatigue (FSS score <4)	29	3.4%	29	3.4%	29	3.4%	30	46.7%
Moderate fatigue (FSS score ≥4 and < 5)	16	6.2%	16	0.0%	14	0.0%	16	68.8%
Severe fatigue (high or severe fatigue (FSS score ≥5)	80	2.5%	80	6.0%	76	5.3%	79	77.2%
P-value^a^		0.591		1.000		1.000		0.010*
FDR Q-value		0.945		1.000		1.000		0.160
Insomnia								
No insomnia post-COVID-19 (0-7)	44	4.5%	44	6.8%	44	2.3%	45	68.9%
Subthreshold insomnia post-COVID-19 (8-14)	48	2.1%	48	4.2%	46	6.5%	48	62.5%
Moderate insomnia post-COVID-19 (15-21)	28	3.6%	28	0.0%	26	3.8%	27	77.8%
Severe insomnia post-COVID-19 (22-28)	6	0.0%	6	0.0%	4	0.0%	6	66.7%
P-value^a^		0.861		0.503		0.866		0.577
FDR Q-value		1.000		0.945		1.000		0.945
Symptoms of anxiety and depression								
Non-cases	33	0.0%	33	3.0%	32	3.1%	33	42.4%
Mild symptoms	21	4.8%	21	4.8%	20	0.0%	22	63.6%
Moderate symptoms	40	2.5%	40	0.0%	39	5.1%	40	77.5%
Severe symptoms	31	6.5%	31	6.5%	28	7.1%	30	90.0%
P-value^a^		0.502		0.355		0.760		0.000*
FDR Q-value		1.000		0.945		0.945		0.000*
Comorbidities								
None	49	4.1	49	14.3	47	10.6	49	65.3
One	43	11.6	43	18.6	40	22.5	44	72.7
Two	19	10.5	19	36.8	19	26.3	19	68.4
Three or more comorbidities	16	0.0	16	6.2	15	13.3	15	73.3
P-value^a^		0.328		0.118		0.308		0.888
FDR Q-value		0.753		0.684		0.753		0.916

a Fisher's exact test.

## 5 Discussion

The present study investigated subjective and objective WM deficits in a selected clinical trial population of individuals with persistent self-reported cognitive difficulties more than six months following COVID-19. Our findings demonstrated that: (1) most individuals exceeded the clinical threshold for subjective WM deficits (BRIEF-A), (2) individuals tended to exceed clinical thresholds on subjective measures more often than on objective measures of WM, and (3) higher symptom levels of anxiety and depression were associated with exceeding clinical thresholds on subjective, but not objective, WM measures.

### 5.1 Subjective working memory deficits

Consistent with the recruitment strategy, which targeted individuals who self-reported cognitive difficulties following SARS-CoV-2 infection, most participants exceeded the clinical threshold for subjective WM deficits, suggesting considerable WM difficulties in everyday life. Interestingly, WM deficits were not influenced by the time since the first infection, hospitalization status, vaccination status, multiple infections, or the severity of symptoms during the acute phase in the current study sample. Nevertheless, the high frequency of exceeding the clinical threshold in the current study also corresponds with numerous studies reporting subjective executive deficits, including WM deficits, in the post-acute phase of COVID-19 (Buer et al., 2024; Cui et al., 2024; Ellingjord-Dale et al., 2024; Hampshire et al., 2024; Søraas et al., 2021).

### 5.2 Objective working memory deficits

Another important finding from the present study is a notable discrepancy between subjective and objective WM measures. Despite high levels of self-reported WM impairment, objective assessments revealed few deficits, with the proportion of participants below the clinical threshold approximating what would be expected based on the normal distribution. The observed discrepancy has also been demonstrated in other studies in the post-COVID-19 patient population and across patient populations with different etiologies, but similar manifestations of executive deficits (Arbula et al., 2024; Cataldo et al., 2024; Haugen et al., 2021; Ingulfsvann Hagen et al., 2023; Øie et al., 2022). However, because post-COVID-19 populations are highly heterogeneous, the extent to which subjective WM deficits are related to objectively measurable WM deficits has not yet been fully clarified, particularly in study samples primarily characterised by self-reported COVID-19 related cognitive deficits.

Arbula et al. (2024) reported that individuals with post-COVID-19 condition frequently reported cognitive difficulties, consistent with their recruitment strategy, whereas standardized neuropsychological tests and experimental cognitive tasks showed limited sensitivity in detecting corresponding objective impairments. Moreover, Arbula et al. (2024) found that participants with post-COVID-19 condition performed worse than controls across objective cognitive measures at the group level, although individual-level impairments were generally mild and usually fell within 1 SD below the normative mean. In the current study, objective WM deficits were defined using a more conservative cutoff of 1.5 SD below the normative mean. While this threshold increases specificity, it may reduce sensitivity to subtler WM deficits that may still contribute to the high prevalence of subjectively reported WM deficits in the current clinical trial population. Bland et al. (2024) found a partial dissociation between subjective and objective cognitive functioning among individuals with post-COVID syndrome, recovered individuals, and COVID-19-naive controls, and also reported that psychological factors were more closely associated with perceived cognitive difficulties than with objective cognitive performance. Taken together, these prior studies provide an important context for the present findings and suggest that subjective cognitive complaints and objective cognitive performance may capture partly distinct aspects of post-COVID-19 cognitive functioning.

Building on these findings, the present study extends prior work by focusing specifically on WM in a selected clinical trial population of individuals with self-reported persistent cognitive difficulties attributed to COVID-19, applying clinical cutoffs to compare subjective and objective WM classifications within the same individuals, and examining whether these classifications vary according to mental health symptoms, fatigue, insomnia, and selected COVID-19-related factors. The findings show that discrepancies between subjective and objective cognitive measures are also evident when WM is examined specifically, and that exceeding the clinical threshold for subjective, but not objective, working memory deficits was associated with symptoms of anxiety and depression. The inclusion of both ecologically valid self-report measures and objective working memory assessments represents a notable strength of the current study. These findings underscore the potential limitations of relying exclusively on performance-based cognitive tests, which may fail to capture real-world WM deficits experienced by individuals following COVID-19. Furthermore, the objective measures included in the current study were relatively brief and may be less capable of capturing WM deficits that could potentially become more evident during sustained cognitive effort. Cognitive fatigability over time on sustained cognitive tasks have previously been demonstrated in the post-acute phase of COVID-19 (Andersson et al., 2025), and may contribute to the discrepancy between subjective WM deficits and objective WM deficits. Thus, future studies should investigate whether sustained cognitive tasks better capture the subjective WM deficits reported in the post-acute phase of COVID-19.

### 5.3 Mental health and working memory deficits

The association between executive deficits and symptoms of ongoing depression and anxiety in the post-acute phase of COVID-19 has not yet been thoroughly investigated (Crivelli et al., 2022; Hampshire et al., 2024; Kirchberger et al., 2023). Our findings showed that subjective WM deficits were more frequent among participants with higher levels of anxiety and depression, highlighting the influence of psychological- and contextual factors on self-reported cognitive impairments. This is consistent with the view that rating-based measures reflect an individual's executive functioning in real-world contexts, sensitive to mental- and physical health status (Friedman et al., 2020). One interpretation of the perceived WM deficits observed in the current study is that they reflect a bidirectional relationship: more severe fatigue and higher levels of anxiety and depression symptoms may increase the perception of WM deficits in everyday life, while these perceived deficits may also exacerbate such symptoms. Prior research supports this notion that psychological factors are more closely related to perceived subjective executive deficits than to objective EF performance (Bland et al., 2024). This interpretation is also consistent with recent transdiagnostic evidence suggesting that mental health factors, including depression and anxiety, are more reliably associated with subjective cognitive complaints than with objective cognitive test performance (Van Patten et al., 2025). This illustrates the complexity in understanding these interactions and the challenge of accounting for individual differences. Clinically, these findings suggest that assessment of anxiety and depression symptoms should be considered when evaluating individuals who report persistent subjective WM difficulties following COVID-19.

The discrepancy between subjective and objective WM measures can also be understood by considering two distinct ways EF is conceptualized and measured (Mareva et al., 2024). While EF can be referred to as a set of cognitive processes enabling flexible thinking, typically assessed using performance-based tasks under controlled conditions, which reflect optimal (state-like) performance (Miyake et al., 2000; Strauss et al., 2006), it also encompasses goal-oriented behavior in daily life, measured through questionnaires like the BRIEF-A, reflecting the use of executive skills in real-world settings, integrating knowledge, beliefs, and values, thus reflecting typical (trait-like) performance (Doebel, 2020). Our study's findings that most individuals exceeded clinical thresholds on subjective WM measures but not on objective measures align with the described distinction between the two types of assessments, underscoring the possibility that each method captures different facets of EF (Mareva et al., 2024; Toplak et al., 2013).

Furthermore, methodological and sample variations may contribute to these discrepancies by causing potential overestimation or underestimation of variability within the patient population. Averaging group scores and comparing them with objective findings can obscure the subjective deficits of individuals who both perceive cognitive difficulties and display deficits in objective tests. This approach could mask clinically relevant discrepancies that might be revealed in a subgroup-level analysis, potentially overlooking patients for whom both subjective and objective measures align. The failure to objectively assess subjective executive deficits has been demonstrated using both mean comparisons and the method of applying clinical cutoffs (Arbula et al., 2024; Cataldo et al., 2024). One characteristic of the present sample is that the majority of participants were highly educated, possibly contributing to the observed discrepancy. A higher cognitive reserve in this sample may limit the ability of objective measures to detect the subtle changes that participants experience (Ariza et al., 2024).

Investigating subjective and objective WM deficits in a patient population such as post-COVID-19 is inherently challenging due to the complexity and heterogeneity of the condition. For instance, some individuals might report subjective complaints primarily associated with fatigue or other various psychological factors, while others might display persistent deficits stemming from substantial cognitive impairments (Bland et al., 2024; Hampshire et al., 2024; Holdsworth et al., 2022; McNeill et al., 2024). The variability within this emerging patient population underscores the need for a comprehensive approach that accounts for individual differences. The complexity of persistent symptoms following COVID-19 calls for an interdisciplinary approach to manage the multisystemic nature of this condition. Additionally, stratifying the population into meaningful subgroups might be necessary to enhance the precision and applicability of research findings (Reese et al., 2023). This approach can be valuable in the development of more individualized prevention and treatment strategies, including non-pharmaceutical management strategies, in the future.

Overall, these findings support the use of comprehensive assessments incorporating both rating-based and performance-based measures when evaluating WM difficulties after COVID-19. The low frequency of objective WM deficits in the current study also underscores the need for test batteries that are sensitive to the cognitive difficulties reported by this patient population. Further research using both subjective and objective measures is needed to clarify which tools best capture clinically relevant cognitive changes and to determine whether interventions targeting fatigue and mental health symptoms may help reduce perceived cognitive difficulties.

## 6 Limitations and generalizability

The current study has several limitations that should be considered when interpreting the findings. First, the cross-sectional design prevents us from drawing causal inferences or conclusions about temporal trajectories of subjective and objective WM deficits following COVID-19. In the absence of cognitive data prior to SARS-CoV-2 infection, we were not able to assess cognitive change. Future studies should consider longitudinal designs and investigate other cognitive domains, such as memory, attention, and EF, using both subjective and objective assessments.

Second, the study population was highly selected. The sample consisted exclusively of individuals who self-reported persistent cognitive difficulties attributed to COVID-19, which limits generalizability to the broader population recovering from COVID-19. Because demographic and clinical information was not collected from individuals excluded during screening because they did not report cognitive difficulties following COVID-19, potential selection bias could not be formally evaluated. Consequently, it remains unclear whether individuals included in the present study differed systematically from those who did not meet the inclusion criteria. The sample also primarily consisted of highly educated women, which may further limit generalizability to more diverse populations. Although subjective WM complaints have also been reported in a larger and more demographically diverse COVID-19 sample (Buer et al., 2024), the demographic composition and selected nature of the present clinical trial population should be considered when interpreting the generalizability of the findings.

Third, the absence of a control group prevents direct comparisons with healthy individuals or other clinical populations, making it difficult to determine whether the observed patterns are specific to post-COVID-19 cognitive dysfunction. The discrepancy observed between subjective and objective WM assessments is also consistent with findings across other clinical groups and may not be unique to post-COVID-19 conditions. As such, this finding should be interpreted as an extension of prior work rather than as a phenomenon specific to post-COVID-19 conditions.

Finally, the available COVID-19-related variables provided limited information about acute illness severity. Although hospitalization status was added as an indicator of acute illness severity, few participants were hospitalized, and clinical data regarding respiratory difficulties or ventilator use were not available. Details regarding timing of the most recent infection, number and severity of comorbid conditions, and performance validity were also limited. These factors may have influenced both subjective reports and objective test outcomes. Furthermore, administering neurocognitive tests remotely introduced limitations, such as the inability to control for distractions in the testing environment. Future studies should include control groups, more diverse samples, better documentation of comorbidities and validity indicators, and broader assessment of acute COVID-19 symptoms and severity.

## 7 Conclusion

This study highlights a low association between subjective and objective WM deficits in a selected clinical trial population of individuals with long-term self-reported cognitive difficulties attributed to COVID-19. Findings also suggest that for some individuals reporting noteworthy executive deficits in the post-acute phase of COVID-19, non-cognitive factors may be influential. These findings should primarily be generalized to individuals with persistent self-reported cognitive difficulties following COVID-19, rather than to the broader population of individuals recovering from COVID-19. Future studies are needed to gain a more in-depth understanding of how COVID-19 impacts EF and WM, and to clarify the underlying mechanisms of these deficits. Understanding the nuanced interplay between cognitive and non-cognitive factors in post-COVID-19 recovery is essential for advancing both clinical assessment and intervention strategies. Future studies should utilize a comprehensive battery of subjective and objective measures to discern which are sensitive enough to detect subtle changes in EF within this newly emerging patient population.

## Funding/support

This study was funded by the 10.13039/501100006095South-Eastern Norway Regional Health Authority (Grant number: 2022/024).

## CRediT authorship contribution statement

**Sofie Buer:** Conceptualization, Data curation, Formal analysis, Writing – original draft, Writing – review & editing. **Bjørn I. Hagen:** Conceptualization, Methodology, Project administration, Resources, Supervision, Writing – review & editing. **Arne Søraas:** Project administration, Writing – review & editing. **Richard A. White:** Formal analysis, Software, Writing – review & editing. **Anners Lerdal:** Conceptualization, Supervision, Writing – review & editing. **Anders B. Nygaard:** Data curation, Formal analysis, Supervision, Writing – review & editing. **Jan Stubberud:** Conceptualization, Funding acquisition, Investigation, Project administration, Supervision, Writing – review & editing.

## Declaration of competing interest

Dr. Søraas reported being an employee and shareholder at Age Labs outside of the submitted work. No other disclosures were reported.

## Data Availability

Data will be made available on request.

## References

[bib1] Altmann D.M., Whettlock E.M., Liu S., Arachchillage D.J., Boyton R.J. (2023). The immunology of long COVID. Nat. Rev. Immunol..

[bib2] Andersson A., Andin J., Levi R., Birberg Thornberg U. (2025). Cognitive performance fatigability, perceived fatigability, and trait fatigue in post-COVID-19 condition: a cross-sectional study. Neuropsychology.

[bib3] Arbula S., Pisanu E., Bellavita G., Menichelli A., Lunardelli A., Furlanis G., Manganotti P., Cappa S., Rumiati R. (2024). Insights into attention and memory difficulties in post-COVID syndrome using standardized neuropsychological tests and experimental cognitive tasks. Sci. Rep..

[bib4] Ariza M., Béjar J., Barrué C., Cano N., Segura B., Bernia J.A., Arauzo V., Balague-Marmaña M., Pérez-Pellejero C., Cañizares S., Muñoz J.A.L., Caballero J., Carnes-Vendrell A., Piñol-Ripoll G., Gonzalez-Aguado E., Riera-Pagespetit M., Forcadell-Ferreres E., Reverte-Vilarroya S., NAUTILUS Project Collaborative Group (2024). Cognitive reserve, depressive symptoms, obesity, and change in employment status predict mental processing speed and executive function after COVID-19. Eur. Arch. Psychiatr. Clin. Neurosci..

[bib5] Baddeley A. (1992). Working memory. Science.

[bib6] Becker J.H., Lin J.J., Twumasi A., Goswami R., Carnavali F., Stone K., Rivera-Mindt M., Kale M.S., Naasan G., Festa J.R., Wisnivesky J.P. (2023). Greater executive dysfunction in patients post-COVID-19 compared to those not infected. Brain Behav. Immun..

[bib7] Bjelland I., Dahl A.A., Haug T.T., Neckelmann D. (2002). The validity of the hospital anxiety and depression scale. J. Psychosom. Res..

[bib8] Bland A.R., Barraclough M., Trender W.R., Mehta M.A., Hellyer P.J., Hampshire A., Penner I.K., Elliott R., Harenwall S. (2024). Profiles of objective and subjective cognitive function in Post-COVID syndrome, COVID-19 recovered, and COVID-19 naïve individuals. Sci. Rep..

[bib9] Buer S., Hagen B.I., Søraas A., White R.A., Bø R., Ellingjord-Dale M., Istre M.S., Brunvoll S.H., Lerdal A., Landrø N.I., Nygaard A.B., Stubberud J. (2024). Executive deficits after SARS-CoV-2 infection: a cross-sectional population study. Brain Behav. Immun. Health.

[bib10] Cataldo S.A., Micciulli A., Margulis L., Cibeyra M., Defeo S., Horovitz S.G., Martino A., Melano R., Mena M., Parisi F., Santoro D., Sarmiento F., Belzunce M.A. (2024). Cognitive impact and brain structural changes in long COVID patients: a cross-sectional MRI study two years post infection in a cohort from Argentina. BMC Neurol..

[bib11] Crivelli L., Calandri I., Corvalán N., Carello M.A., Keller G., Martínez C., Arruabarrena M., Allegri R. (2022). Cognitive consequences of COVID-19: results of a cohort study from South America. Arquivos de Neuro-Psiquiatria.

[bib12] Cui R., Gao B., Ge R., Li M., Li M., Lu X., Jiang S. (2024). The effects of COVID-19 infection on working memory: a systematic review. Curr. Med. Res. Opin..

[bib13] Davis H.E., McCorkell L., Vogel J.M., Topol E.J. (2023). Long COVID: major findings, mechanisms and recommendations. Nat. Rev. Microbiol..

[bib14] Doebel Sabine (2020). Rethinking executive function and its development. Perspect. Psychol. Sci..

[bib15] Douaud G., Lee S., Alfaro-Almagro F., Arthofer C., Wang C., McCarthy P., Lange F., Andersson J.L.R., Griffanti L., Duff E., Jbabdi S., Taschler B., Keating P., Winkler A.M., Collins R., Matthews P.M., Allen N., Miller K.L., Nichols T.E., Smith S.M. (2022). SARS-CoV-2 is associated with changes in brain structure in UK biobank. Nature.

[bib16] Ellingjord-Dale M., Brunvoll S.H., Søraas A. (2024). Prospective memory assessment before and after Covid-19. N. Engl. J. Med..

[bib17] Fernandez-de-las-Peñas C., Notarte K.I., Macasaet R., Velasco J.V., Catahay J.A., Ver A.T., Chung W., Valera-Calero J.A., Navarro-Santana M. (2024). Persistence of post-COVID symptoms in the general population two years after SARS-CoV-2 infection: a systematic review and meta-analysis. J. Infect..

[bib18] Friedman Naomi P., Hatoum Alexander S., Gustavson Daniel E., Corley Robin P., Hewitt John K. (2020). Executive functions and impulsivity are genetically distinct and independently predict psychopathology: results from two adult twin studies. Clin. Psychol. Sci..

[bib19] Gerst E.H., Cirino P.T., Fletcher J.M., Yoshida H. (2017). Cognitive and behavioral rating measures of executive function as predictors of academic outcomes in children. Child Neuropsychol..

[bib20] Hagen B.I., Lerdal A., Søraas A., Landrø N.I., Bø R., Småstuen M.C., Becker J., Stubberud J. (2022). Cognitive rehabilitation in post-COVID-19 condition: a study protocol for a randomized controlled trial. Contemp. Clin. Trials.

[bib21] Hampshire A., Azor A., Atchison C., Trender W., Hellyer P.J., Giunchiglia V., Husain M., Cooke G.S., Cooper E., Lound A., Donnelly C.A., Chadeau-Hyam M., Ward H., Elliott P. (2024). Cognition and memory after Covid-19 in a large community sample. N. Engl. J. Med..

[bib22] Hampshire A., Trender W., Chamberlain S.R., Jolly A.E., Grant J.E., Patrick F., Mazibuko N., Williams S.C., Barnby J.M., Hellyer P., Mehta M.A. (2021). Cognitive deficits in people who have recovered from COVID-19. EClinicalMedicine.

[bib23] Haugen I., Stubberud J., Ueland T., Haug E., Øie M.G. (2021). Executive dysfunction in schizophrenia: predictors of the discrepancy between subjective and objective measures. Schizophr. Res.: Cognition.

[bib24] Holdsworth D.A., Chamley R., Barker-Davies R., O'Sullivan O., Ladlow P., Mitchell J.L., Dewson D., Mills D., May S.L.J., Cranley M., Xie C., Sellon E., Mulae J., Naylor J., Raman B., Talbot N.P., Rider O.J., Bennett A.N., Nicol E.D. (2022). Comprehensive clinical assessment identifies specific neurocognitive deficits in working-age patients with long-COVID. PLoS One.

[bib25] Øie M.G., Rødø A.S.B., Bølgen M.S., Pedersen M., Asprusten T.T., Wyller V.B.B. (2022). Subjective and objective cognitive function in adolescent with chronic fatigue following Epstein-Barr virus infection. J. Psychosom. Res..

[bib26] Ingulfsvann Hagen B., Landrø N.I., Hoorelbeke K., Lau B., Stubberud J. (2023). Characteristics associated with the discrepancy between subjective and objective executive functioning in depression. Appl. Neuropsychol.: Adult.

[bib27] Johansson S., Kottorp A., Lee K.A., Gay C.L., Lerdal A. (2014). Can the fatigue severity scale 7-item version be used across different patient populations as a generic fatigue measure—A comparative study using a rasch model approach. Health Qual. Life Outcome.

[bib28] Karlsen R.H., Karr J.E., Saksvik S.B., Lundervold A.J., Hjemdal O., Olsen A., Iverson G.L., Skandsen T. (2022). Examining 3-month test-retest reliability and reliable change using the Cambridge neuropsychological test automated battery. Appl. Neuropsychol.: Adult.

[bib29] Kirchberger I., Peilstöcker D., Warm T.D., Linseisen J., Hyhlik-Dürr A., Meisinger C., Goßlau Y. (2023). Subjective and objective cognitive impairments in non-hospitalized persons 9 months after SARS-CoV-2 infection. Viruses.

[bib30] Lerdal A., Kottorp A., Gay C., Aouizerat B.E., Portillo C.J., Lee K.A. (2011). A 7-item version of the fatigue severity scale has better psychometric properties among HIV-infected adults: an application of a rasch model. Qual. Life Res..

[bib31] Lezak M.D., Howieson D.B., Bigler E.D., Tranel D. (2012).

[bib32] Lyngstad T.M. (2022). https://www.fhi.no/contentassets/8a971e7b0a3c4a06bdbf381ab52e6157/vedlegg/3.-alle-ukerapporter-2021/ukerapport-uke-52-27.12.21---02.01.22.pdf.

[bib33] Mareva S., Holmes J., Lead Investigators, Astle D., Baker K., Gathercole S., Holmes J., Kievit R., Manly T., Akarca D., Bathelt J., Bettencourt M., Bennett M., Bignardi G., Bishop S., Bottacin E., Bridge L., Brkic D., Team Of Researchers And PhD Students (2024). Mapping neurodevelopmental diversity in executive function. Cortex.

[bib34] McNeill R., Marshall R., Fernando S.A., Harrison O., Machado L. (2024). COVID-19 may enduringly impact cognitive performance and brain haemodynamics in undergraduate students. Brain Behav. Immun..

[bib35] Miyake A., Friedman N.P., Emerson M.J., Witzki A.H., Howerter A., Wager T.D. (2000). The Unity and diversity of executive functions and their contributions to complex “Frontal Lobe” tasks: a latent variable analysis. Cogn. Psychol..

[bib36] Morin C.M., Belleville G., Bélanger L., Ivers H. (2011). The insomnia severity index: psychometric indicators to detect insomnia cases and evaluate treatment response. Sleep.

[bib37] Nin V., Delgado H., Muniz‐Terrera G., Carboni A. (2022). Partial agreement between task and BRIEF‐P‐based EF measures depends on school socioeconomic status. Dev. Sci..

[bib38] O'Mahoney L.L., Routen A., Gillies C., Ekezie W., Welford A., Zhang A., Karamchandani U., Simms-Williams N., Cassambai S., Ardavani A., Wilkinson T.J., Hawthorne G., Curtis F., Kingsnorth A.P., Almaqhawi A., Ward T., Ayoubkhani D., Banerjee A., Calvert M. (2023). The prevalence and long-term health effects of Long Covid among hospitalised and non-hospitalised populations: a systematic review and meta-analysis. eClinicalMedicine.

[bib39] Orfei M.D., Porcari D.E., D'Arcangelo S., Maggi F., Russignaga D., Ricciardi E. (2022). A new look on Long-COVID effects: the functional brain fog syndrome. J. Clin. Med..

[bib40] Pickett I. (2021). COVID-19 cognitive assessment battery. Cambridge Cognit..

[bib41] Poletti S., Palladini M., Mazza M.G., De Lorenzo R., Irene B., Sara B., Beatrice B., Ceciclio B., Stefania C., Valentina C., Elisa C., Jacopo C., Marta C., Elena C., Federica C., Sarah D., Greta D., Camilla D.P., The COVID-19 BioB Outpatient Clinic Study group (2022). Long-term consequences of COVID-19 on cognitive functioning up to 6 months after discharge: role of depression and impact on quality of life. Eur. Arch. Psychiatr. Clin. Neurosci..

[bib42] Reese J.T., Blau H., Casiraghi E., Bergquist T., Loomba J.J., Callahan T.J., Laraway B., Antonescu C., Coleman B., Gargano M., Wilkins K.J., Cappelletti L., Fontana T., Ammar N., Antony B., Murali T.M., Caufield J.H., Karlebach G., McMurry J.A. (2023). Generalisable long COVID subtypes: findings from the NIH N3C and RECOVER programmes. EBioMedicine.

[bib43] Reid K.J., Ingram L.T., Jimenez M., Orban Z.S., Abbott S.M., Grimaldi D., Knutson K.L., Zee P.C., Koralnik I.J., Maas M.B. (2024). Impact of sleep disruption on cognitive function in patients with postacute sequelae of SARS-CoV-2 infection: initial findings from a Neuro-COVID-19 clinic. Sleep Adv..

[bib44] Roth R.M., Isquith P.K., Gioia G.A. (2005). Behavior rating inventory of executive function®—adult version.

[bib45] Snyder H.R., Friedman N.P., Hankin B.L. (2021). Associations between task performance and self-report measures of cognitive control: shared versus distinct abilities. Assessment.

[bib46] Soto E.F., Kofler M.J., Singh L.J., Wells E.L., Irwin L.N., Groves N.B., Miller C.E. (2020). Executive functioning rating scales: ecologically valid or construct invalid?. Neuropsychology.

[bib47] Søraas A., Bø R., Kalleberg K.T., Støer N.C., Ellingjord-Dale M., Landrø N.I. (2021). Self-reported memory problems 8 months after COVID-19 infection. JAMA Netw. Open.

[bib48] Søraas A., Grødeland G., Granerud B.K., Ueland T., Lind A., Fevang B., Murphy S.L., Huse C., Nygaard A.B., Steffensen A.K., al-Baldawi H., Holberg-Petersen M., Andresen L.L., Ågnes C., Ranheim T., Schanke Y., Istre M., Dahl J.A., Chopra A. (2022). Breakthrough infections with the omicron and delta variants of SARS-CoV-2 result in similar re-activation of vaccine-induced immunity. Front. Immunol..

[bib49] Stavem K., Einvik G., Tholin B., Ghanima W., Hessen E., Lundqvist C. (2022). Cognitive function in non-hospitalized patients 8–13 months after acute COVID-19 infection: a cohort study in Norway. PLoS One.

[bib50] Stern A.F. (2014). The hospital anxiety and depression scale. Occup. Med..

[bib51] Strauss E., Sherman E.M.S., Spreen O. (2006). A Compendium of Neuropsychological Tests: Administration, Norms, and Commentary.

[bib52] Toplak M.E., West R.F., Stanovich K.E. (2013). Practitioner review: do performance‐based measures and ratings of executive function assess the same construct?. JCPP (J. Child Psychol. Psychiatry).

[bib53] Van Aken B.C., Rietveld R., Wierdsma A.I., Voskes Y., Pijnenborg G.H.M., Van Weeghel J., Mulder C.L. (2023). Self-report versus performance based executive functioning in people with psychotic disorders. Schizophr. Res.: Cognition.

[bib54] Van Patten R., Mulhauser K., Austin T.A., Bellone J.A., Cotton E., Chan L., Twamley E.W., Sawyer K., LaFrance W.C. (2025). The association between subjective and objective cognitive functioning from a transdiagnostic perspective: an umbrella review and meta-analysis. Clin. Psychol. Rev..

[bib55] Waid-Ebbs J.K., Wen P.-S., Heaton S.C., Donovan N.J., Velozo C. (2012). The item level psychometrics of the behaviour rating inventory of executive function-adult (BRIEF-A) in a TBI sample. Brain Inj..

[bib56] Wechsler D. (2008).

[bib57] Wong A., Nyenhuis D., Black S.E., Law L.S.N., Lo E.S.K., Kwan P.W.L., Au L., Chan A.Y.Y., Wong L.K.S., Nasreddine Z., Mok V. (2015). Montreal cognitive assessment 5-Minute protocol is a brief, valid, reliable, and feasible cognitive screen for telephone administration. Stroke.

[bib58] Zigmond A.S., Snaith R.P. (1983). Hospital anxiety and depression scale.

