# Supplementary material for: Subjective and objective working memory deficits in the post-acute phase of COVID-19 in a clinical trial population
Source: Brain Behav Immun Health. 2026 Jun 4;55:101278. doi: 10.1016/j.bbih.2026.101278 (PMC13272546; doi:10.1016/j.bbih.2026.101278)
Supplement: Multimedia component 1 [file mmc1.docx]

**Supplement 1**

**Subjective and Objective Working Memory Deficits in the post-acute phase of COVID-19 in a Clinical Trial Population**

Table of Contents

[eSupplementary Figure 1. First time SARS-CoV-2 Positive Date Range for the patient population 2](#_Toc229402753)

[eSupplementary Table 1. Mean *T*-scores, range and proportion (%) scoring above clinical cut-across on the BRIEF-A 3](#_Toc229402754)

[eSupplementary Figure 3. Frequency above clinical threshold by sex 5](#_Toc229402755)

[eSupplementary Figure 4. Frequency above clinical threshold by hospitalization status 5](#_Toc229402756)

## eSupplementary Figure 1. First time SARS-CoV-2 Positive Date Range for the patient population


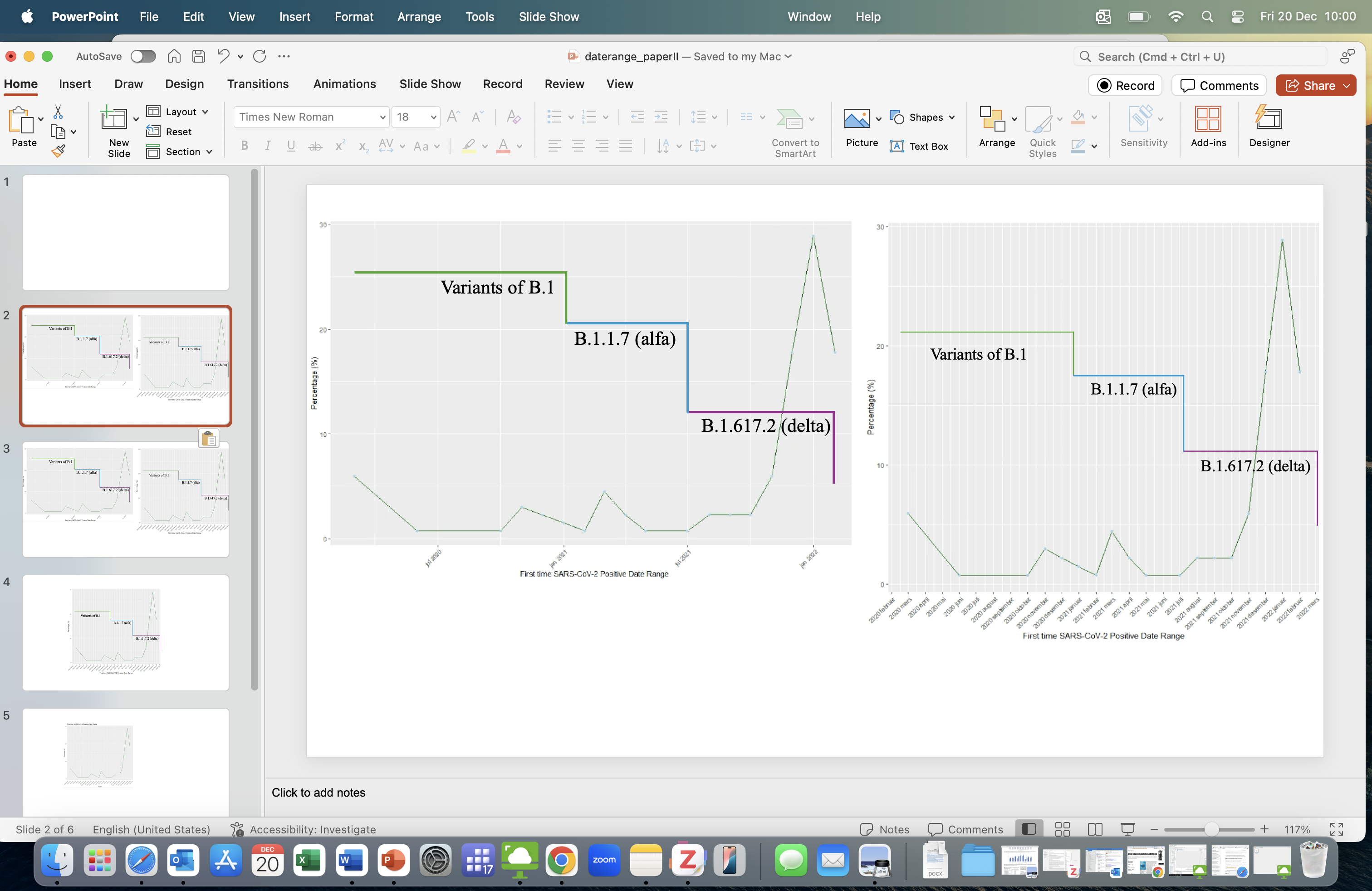


Figure 1 First time SARS-CoV-2 positive date range for the patient population. Approximately 50% of all sequenced samples in [Masked ]are confirmed variants of B.1 in the period of February 2020 to January 2021, B.1.1.7 (alfa) for the period of February 2021 to July 2021 and B.1.617.2 (delta) for the period of July 2021 to September 2021 from the published report for week 52, period between 27.12.2021-02.01.2022 on Covid-19 by (Lyngstad, 2022).

## eSupplementary Table 1. Mean *T*-scores, range and proportion (%) scoring above clinical cut-across on the BRIEF-A

Table 1. Mean T-scores, range and proportion (%) scoring above clinical cut-off (T ≥ 65) across all indexes and subscales of the BRIEF-A (n=129)

| BRIEF-A Indexes and subscales  (N=129) | *n* | Mean T score (*SD*) | Range | % over cutoffs |
| --- | --- | --- | --- | --- |
| Global Executive Composite (GEC) | 117 | 59.8 (10.7) | 39-95 | 32 |
| Metacognition Index (MI) | 122 | 62.6 (11.0) | 37-95 | 47 |
| Behavioral Regulation Index (BRI) | 123 | 54.3 (10.5) | 36-89 | 22 |
| Emotional control | 127 | 54.1 (11.4) | 38-90 | 17 |
| Self-monitor | 127 | 50.1 (10.8) | 37-87 | 9 |
| Initiate | 126 | 61.4 (12.6) | 38-94 | 43 |
| Shift | 129 | 55.4 (9.60) | 39-81 | 18 |
| Working memory | 128 | 69.6 (11.1) | 43-94 | 69 |
| Inhibition | 127 | 53 (10.1) | 37-83 | 16 |
| Plan Organize | 127 | 59.7 (11.0) | 38-91 | 39 |
| Task Monitor | 128 | 60.7 (11.2) | 38-93 | 38 |
| Organization of Materials | 128 | 54.5 (12.1) | 36-86 | 22 |
|  |  |  |  |  |

eSupplementary Figure 2. Proportion (%) of participants scoring above the clinical cut-off on BRIEF-A by gender


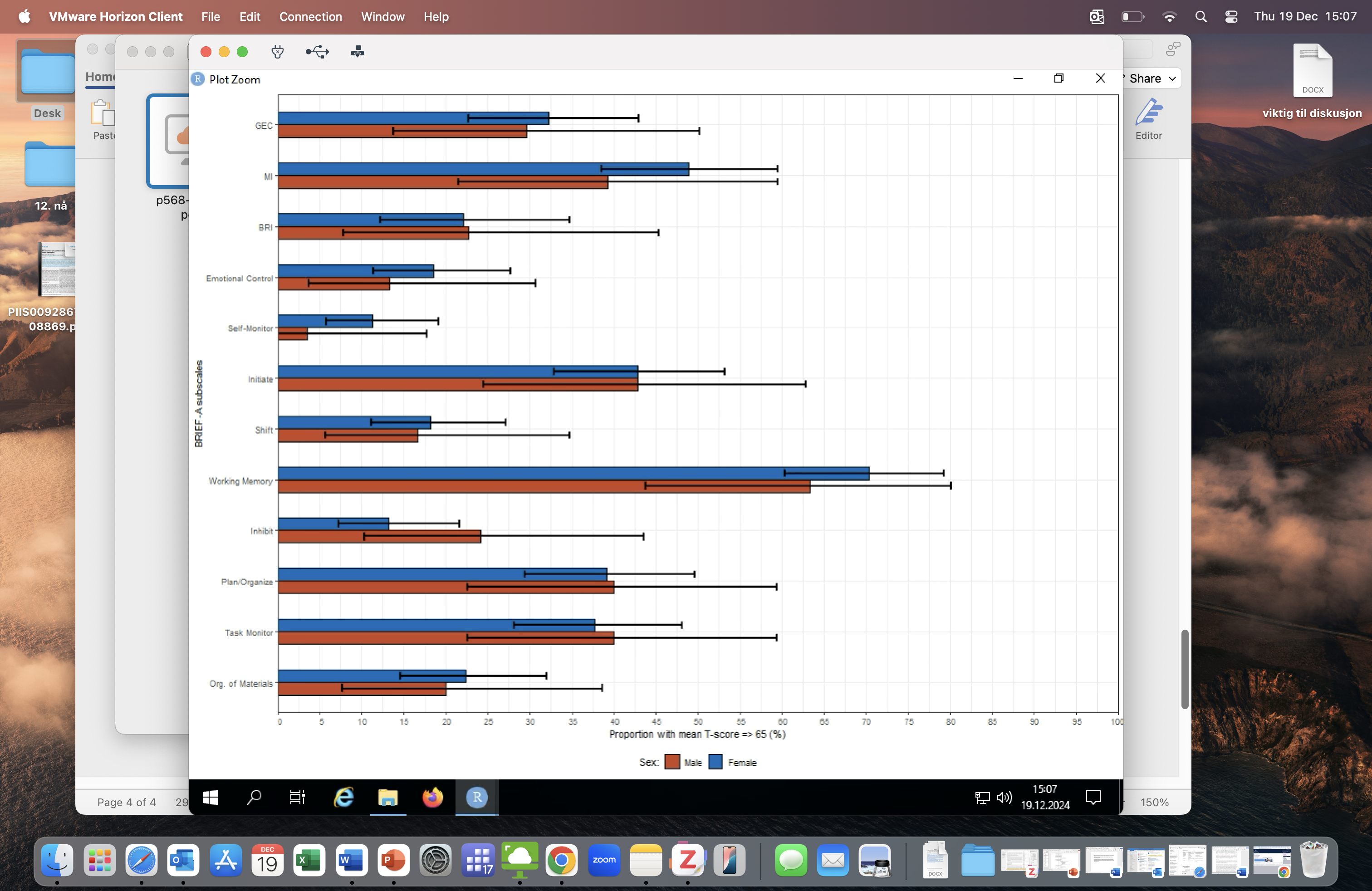


Figure 2 Proportion (%) of participants scoring above the clinical cut-off on BRIEF-A by gender

## eSupplementary Figure 3. Frequency above clinical threshold by sex


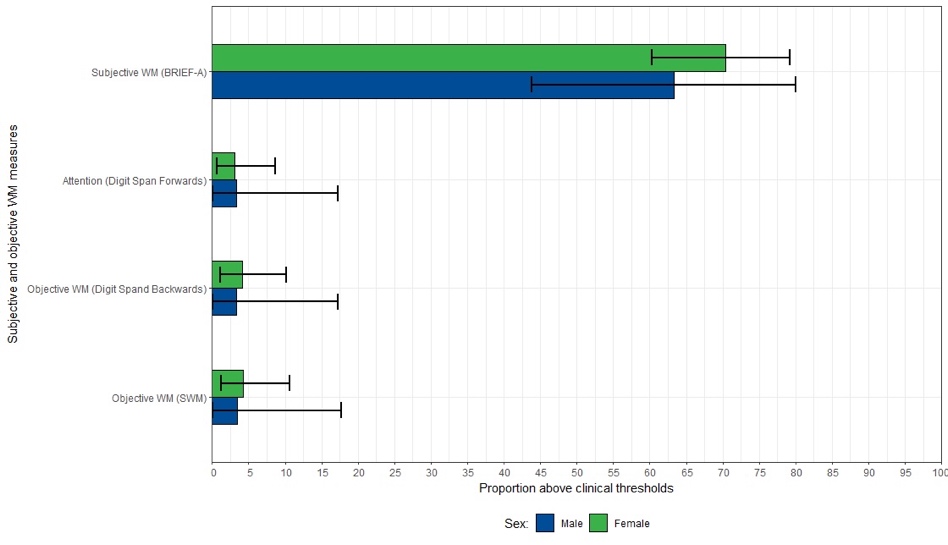


The proportion (%) of study participants scoring above the clinical cutoff (T ≥65) for subjective WM deficits and 1 *SD* below normative mean for objective attention and WM, displayed similar trends across sexes. Fewest deficits were observed for objective attention (6.7% vs. 7.1%). For objective WM deficits the differences were 20% vs. 17% (WAIS-IV) and 21% vs. 16% (SWM). The larges numerical differences were observed for subjective WM deficits, with 63% vs. 70%. Males more frequently exhibited objective WM deficits, whereas females showed more subjective WM deficits (Figure 3). However, none of these differences were statistically significant.

## eSupplementary Figure 4. Frequency above clinical threshold by hospitalization status


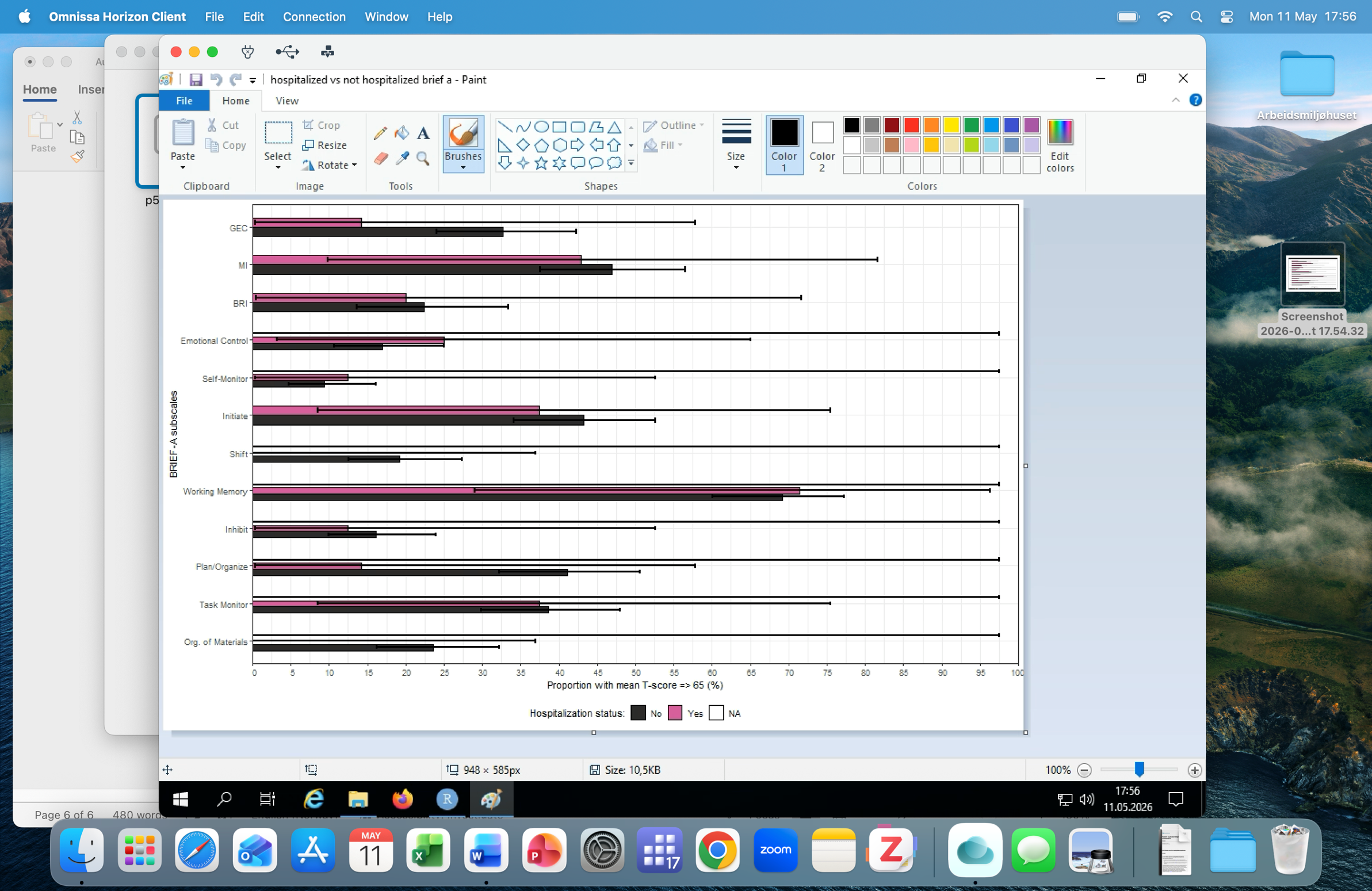


Similar patterns were observed for hospitalized and non-hospitalized participants on self-reported executive functioning difficulties exceeding the clinical threshold. However, the small number of hospitalized participants limited the ability to detect potentially meaningful group differences.
